# Interfering with Tumor Hypoxia for Radiotherapy Optimization

**DOI:** 10.1186/s13046-021-02000-x

**Published:** 2021-06-21

**Authors:** Irma Telarovic, Roland H. Wenger, Martin Pruschy

**Affiliations:** 1 Laboratory for Applied Radiobiology, Department of Radiation Oncology, University Hospital Zurich, University of Zurich, Raemistrasse 100, 8091 Zurich, Switzerland; 2 Institute of Physiology, University of Zurich, Winterthurerstrasse 190, 8057 Zurich, Switzerland

**Keywords:** hypoxia, radiotherapy, SBRT, radiosensitizers

## Abstract

Hypoxia in solid tumors is an important predictor of treatment resistance and poor clinical outcome. The significance of hypoxia in the development of resistance to radiotherapy has been recognized for decades and the search for hypoxia-targeting, radiosensitizing agents continues. This review summarizes the main hypoxia-related processes relevant for radiotherapy on the subcellular, cellular and tissue level and discusses the significance of hypoxia in radiation oncology, especially with regard to the current shift towards hypofractionated treatment regimens. Furthermore, we discuss the strategies to interfere with hypoxia for radiotherapy optimization, and we highlight novel insights into the molecular pathways involved in hypoxia that might be utilized to increase the efficacy of radiotherapy.

## Background

Solid tumors require the formation of a neovascular network in order to grow and survive [1, 2]. Rapidly proliferating tumor cells create a hypoxic microenvironment that induces tumor angiogenesis. The rapidly growing blood vessels comprising the neovascular network are often aberrant, disorganized and dysfunctional, leading to an inadequate oxygen supply which further increases tumor hypoxia [3, 4]. Oxygen demand, on the other hand, is often deregulated subsequent to tumor-specific metabolic changes. Together, this results in a dynamic, rapidly changing tumor microenvironment characterized by regions of nutrient deprivation and oxygen deficiency [5–7]. Tumor hypoxia was recognized as early as in the 1950s, when Gray and Thomlinson demonstrated by mathematical calculations, based on histological sections of human tumors, that regions around necrotic areas display a hypoxic oxygen gradient [8]. Studies in the following decades identified oxygen deficiency to be characteristic for the majority of solid human tumors [9–15]. In contrast to the early point of view that a radial hypoxia gradient primarily expands from the necrotic tumor core towards the tumor rim, it has since been established that a wide range of fluctuating oxygen tension values (from mild oxygen deficiency to anoxia) are heterogeneously dispersed in the tumor microenvironment. Both hypoxia and necrosis were independently proven to be predictors of poor clinical outcome, independent of the tumor stage, histological grade and lymph node status [16–18]. Hypoxia has also been associated with genomic instability [19], immune suppression [20, 21], the different steps of the metastatic cascade (including invasion, migration, intravasation and extravasation, formation and support of the premetastatic niche) [22], and an increase in resistance to chemotherapy and radiotherapy [23, 24].

In this review, we focus on the role of hypoxia for the treatment response to radiotherapy and discuss existing and emerging strategies to overcome hypoxia-induced radioresistance.

### The Definition of Hypoxia

Hypoxia is generally defined as a reduced partial pressure of oxygen (i.e. pO_2_ or oxygen tension) below a physiological level. However, attributing a single number to this definition is not so straightforward: physiological pO_2_ in the human body ranges from ~ 100 mmHg in the lung alveoli to values below 1 mmHg in the mitochondria. This pressure gradient or the “oxygen cascade” ensures the diffusion of oxygen throughout the body, guiding it from the “supplier” (the alveolar gas) to the “sinks” (the cells). Physiologically, all values of pO_2_ present in this cascade could be considered “normoxic” [25].

In a recent review [26], Rey et al. calculated the median oxygen tension in various tissues based on previous publications. The oxygen tension in normal tissues ranged from 30 to 52 mmHg (corresponds to 3.9 to 6.8% oxygen concentration in the gas phase at sea level), while tumoral oxygen tension ranged in between 5.3 to 14 mmHg (0.7 to 1.8%), often with a considerable fraction of cells reaching below 5 mmHg (0.7%) [27]. Although these median values would suggest an overall hypoxic environment in most tumors, the typical tumoral pattern of oxygenation is highly heterogenous and dynamic. Such heterogeneity is a result of the distinct organization, structure and function of the tumor vasculature and ongoing angiogenic processes. In contrast to normal blood vessel networks, the tumor vasculature comprises a chaotic network of aberrant, hierarchically disorganized and often dysfunctional vessels [3, 4]. As a result, two types of hypoxia can be described in the tumor tissue [28]. Chronic or diffusion-limited hypoxia arises from the oxygen diffusion limit of approx. 100 μm and is therefore relevant for cells that do not have access to a blood vessel within this limiting distance. Acute or perfusion-limited hypoxia, on the other hand, is a result of temporary instabilities in the blood flow. Taken together, this illustrates the complexity and heterogeneity of the oxygenation status in solid tumors. Consequently, reducing the definition of hypoxia to a specific oxygen level is not possible without a specific context, which needs to take into account the tissue type and cellular composition, the spatiotemporal heterogeneity of oxygen tension in the tumor and most importantly, the endpoint of interest.

## Radiotherapy and Tumor Hypoxia

The first indications that a tissue poorly supplied with oxygen might be more resistant to ionizing radiation (IR) came about as early as in the 1920s [29, 30], culminating in Gray’s seminal paper in 1953 [31], where he demonstrated on the preclinical level that a decrease in hypoxia, achieved by improving oxygen delivery, results in increased radiosensitivity. These discoveries paved the way to numerous subsequent studies, focusing on elucidating the underlying mechanisms of hypoxia-induced radioresistance, on determining the significance of hypoxia-induced radioresistance on the clinical level, and finally, on developing strategies for radiosensitization.

### Direct Interaction of Ionizing Radiation with Oxygen: the Oxygen Fixation Hypothesis

**Fig. 1:**
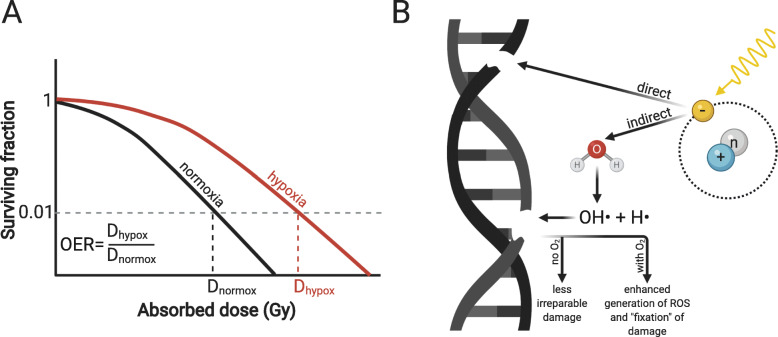
Direct interaction of ionizing radiation with oxygen: the oxygen fixation hypothesis. (**a**) Oxygen enhancement ratio (OER). Curves represent the surviving fractions of cells irradiated with increasing doses of ionizing radiation under normoxic (black) and hypoxic (red) conditions. The OER is defined as the ratio of the radiation dose under hypoxia and the radiation dose under normoxia to achieve the same biological effect. (**b**) The oxygen fixation hypothesis mechanistically describes the role of oxygen for enhanced DNA damage and cell killing under normoxic conditions and includes the initial formation of more ROS and chemical derivatization (“fixation”) of DNA damage in presence of oxygen. See details in text

The dependency of cellular responses to IR on the oxygen level has been recognized for almost a century [30, 31]. This dependency is quantitatively described as the oxygen enhancement ratio (OER), defined as the ratio between the radiation dose under hypoxic conditions and the radiation dose under normoxic conditions necessary to achieve the same amount of cell killing (determined *in vitro* by clonogenic assays) (Fig. 1a). For most cells in culture, the OER between anoxia (i.e. complete oxygen deprivation during irradiation) and air (150 mmHg or 21% oxygen) lies between 2.5 and 3 [32]. In a study measuring the change in OERs over a range of oxygen tension values, Wouters et al. [33] discovered that the greatest change in radiosensitivity i.e. the highest OER occurs in the range of 0.5–20 mmHg (0.05–2.5%). with an increase above 20 mmHg only showing minor changes in radiosensitivity. Together with the physiological values stated above, it can be concluded that from a radiobiological point of view, most normal tissues under physiological conditions are sufficiently oxygenated. On the other hand, for most tumors, a significant proportion of tumor cells are exposed to hypoxic conditions and consequently are prone to radioresistance.

The mechanism behind the oxygen-driven increase of cellular radiosensitivity is explained by the “oxygen fixation hypothesis” (Fig. 1b) [29, 32]. In short, this hypothesis states that the probability of permanent IR-induced DNA damage is higher in the presence than in the absence of oxygen. Upon impinging on the tissue, photons ionize the atoms of the absorbing material and generate free electrons. These electrons mediate the IR-induced damage, either by directly damaging the macromolecules, or by interacting with water in a process that yields hydroxyl (OH•) and hydrogen (H•) radicals. These short-lived and highly reactive molecules react with and damage the macromolecules. In absence of oxygen, the extent of these reactions is limited due to the radicals’ instability, and the majority of the damage is readily repaired. In presence of oxygen, additional reactive oxygen species (ROS) are formed, e.g. hydrogen peroxides, which increase the overall concentration of DNA damaging agents. Furthermore, free radicals produced in the critical targets (R•) can also react with oxygen to produce first peroxyl radicals (ROO•) and ultimately the more stable and less well repairable ROOH, thereby chemically “fixing” the damage. This phenomenon, also known as the “Sauerstoffeffekt” (i.e. oxygen effect), forms the basis of our understanding of oxygen-mediated changes in radiosensitivity. Notably, the oxygen effect is by definition a purely physicochemical effect and does not depend on the radiobiological processes explained below.

### Hypoxia-Induced Processes Relevant for Radiotherapy

In the physiological state, oxygen and nutrient depletion constitute a major source of cellular stress and thereby induce evolutionary conserved adaptive changes that either increase the delivery or decrease the consumption of missing substances [34, 35]. An increase in erythropoiesis and angiogenesis [36, 37], in epithelial permeability [38] and vascular tone [39] may all contribute to enhance the delivery of oxygen to the hypoxic tumor area. To attenuate oxygen consumption, hypoxic cells decrease oxidative phosphorylation by switching to anaerobic glycolysis [40] and reduce the number of mitochondria (i.e. mitophagy) [41]. Furthermore, to facilitate tissue recovery after potential damage induced under such circumstances, hypoxia can induce expression of growth factors and thereby promote cell proliferation and survival [42]. In case these compensatory actions fail and severe hypoxia persists, normal cells undergo cell cycle arrest and eventually cell death [43–45].

Many of the aforementioned mechanisms, such as induction of angiogenesis and promotion of cell proliferation, remain active in the tumor. However, unlike in healthy cells, selective pressure of hypoxia-induced cell cycle arrest and apoptosis leads to a selection of more aggressive malignant clones with disruptions in physiological feedback mechanisms that would normally halt uncontrolled cellular growth [42, 46, 47]. As a result, hypoxia-induced adaptive changes effectively promote cancer progression. In addition to the listed physiological mechanisms that cancer cells co-opt, hypoxia directly promotes immortalization [48], genetic instability [7, 19], immune evasion [20, 21], cancer stem cell survival [49], invasion and metastasis [22]. All of these mechanisms, but especially the selection of malignant, apoptosis-resistant clones, regulation of angiogenesis and vasculogenesis, metabolic changes and interference with the DNA damage response, determine hypoxia as one of the key factors that mediate resistance to radiotherapy, beyond the oxygen effect. In this review, we discuss the molecular processes behind these radiotherapy resistance-promoting mechanisms. A detailed overview of the complex processes through which hypoxia promotes malignancy is not the scope this review and the reader is referred to other comprehensive literature [6, 26, 50, 51].

#### Oxygen Sensing Mechanisms: Hypoxia-Inducible Factors and Beyond

Hypoxia-inducible factors (HIFs) constitute a family of transcription factors that act as the main mediators of the cellular response to hypoxia [34, 36, 51–56] and were first identified almost three decades ago as the key regulators of hypoxia-induced erythropoietin transcription [57]. HIFs are heterodimers formed by a constitutively expressed β-subunit and an oxygen-sensitive α-subunit. In mammals, three isoforms of the α-subunit differentiate the three paralogs: HIF-1α, HIF-2α and HIF-3α. Upon binding of the HIF-β subunit, these three paralogs compose the three transcription factors HIF-1, HIF-2 and HIF-3, respectively. In this review, we focus on HIF-1 as the most investigated isoform in the context of radiotherapy optimization and only mention the emerging data relating to HIF-2 and radiotherapy. The role of HIF-3 remains elusive and as such, is not covered in this review.

In the presence of oxygen, the α-subunit of HIF-1 is posttranslationally hydroxylated through the interaction with oxygen-sensitive dioxygenases, namely the HIF prolyl-4-hydroxylase domain enzymes (PHDs) [58–60]. Hydroxylation allows for binding of the von Hippel-Lindau (VHL) tumor suppressor protein. In this bound state, the α-subunit is targeted for poly-ubiquitination and proteasomal degradation. PHDs require oxygen as a cofactor for activity and are therefore rendered inactive when oxygen is not available. Consequently, in hypoxic conditions HIF-1α and HIF-2α remain stable and bind to the constitutively expressed HIF-β subunit, forming the transcriptionally active heterodimeric HIF-1 and HIF-2 complexes, respectively, which bind hypoxia response elements (HREs) in the regulatory regions of hundreds of target genes [61, 62]. In contrast to this direct binding to the regulatory regions and subsequent gene activation, HIF-1/2 dependent transcriptional modulation can also occur indirectly, through transactivation of genes for long non-coding and micro RNAs [62, 63] as well as DNA and histone modifiers [64]. Through a complex interaction between direct and indirect effects, HIFs modulate the expression of over thousand genes and regulate the majority of the hypoxia-induced adaptive mechanisms (reviewed in e.g. [50, 60, 61, 65]).

The majority of studies over the last decades, that investigated hypoxia-altered gene expression, focused on the HIF-pathway. However, Jumonji-family of histone demethylases has long been speculated to mediate also HIF-independent gene expression regulation [66]. In 2019 Batie et al. [67] and Chakraborty et al. [68] clearly demonstrated for the first time direct, HIF-independent oxygen-mediated gene expression modulation by the lysine-specific Jumonji-family demethylases KDM5A and KDM6A. Moreover, cellular phenotype alterations under low pO_2_ conditions also occur due to numerous adaptive mechanisms other than direct gene expression control. For example, oxygen availability affects mRNA transcription, splicing, stability and translation, protein folding, protein stability and enzymatic activity [32, 50]. Furthermore, metabolic disturbances and other determinants of cellular stress that accompany hypoxia interfere with both HIF-dependent and HIF-independent signaling networks. This includes for example the nutrient-sensing PI3K/AKT/mTOR pathway [69] and the unfolded protein response (UPR) [70]. Overall, the research into the HIF-independent effects of hypoxia is ongoing, and the potential role of such effects for hypoxia-induced radioresistance remain to be investigated. For a comprehensive overview, the reader is referred to [35].

Given the role of HIF-1 as a key player for tumor angiogenesis and as part of the cellular responses to cancer treatments, it is not surprising that the status of HIF-1α overexpression correlates with poor prognosis in many cancer types (head and neck, oesophagus, pancreas, stomach, gall bladder, liver, colon, lung, pleura, breast, ovaries, uterus and bladder [71–84]). Similar to HIF-1α, HIF-2α overexpression also correlates with poor prognosis in several cancer types (head and neck, brain, liver, colon, lung, bladder, kidney) [85–91]. Thus, next to the direct physicochemical role of a low pO_2_ for radiotherapy resistance (see above), HIF-1 mediates biological processes relevant for hypoxia-related radiotherapy resistance. It is important to note that the transcriptionally active level of HIF-1 starts to exponentially increase already below the oxygen tension of 40 mmHg (6%) [92, 93]. Thus, compared to the physicochemical “oxygen effect” and “radiobiological hypoxia” described above, which occur in the range of 0.5 to 20 mmHg (0.05 to 2.5%), HIF-1-mediated biological effects are exerted at a significant earlier stage of oxygen deprivation.

#### The Interaction of Radiotherapy and HIF-1

The relationship between HIF-1 and radiotherapy is complex and bilateral. On the one hand, HIF-1 confers radioresistance through various cancer-promoting mechanisms. On the other hand, radiotherapy activates HIF-1 and its downstream targets, both through hypoxia-dependent and -independent mechanisms.

##### Radiotherapy-Induced HIF-1 Activation

Hypoxia-dependent radiotherapy-induced HIF-1 activation in the tumor occurs primarily through tumor vascular damage and subsequent oxygen deprivation while multiple mechanisms contribute to hypoxia-independent RT-induced HIF-1 activation. The importance of such mechanisms for radiotherapy was undoubtedly demonstrated by Moeller et al. [94], who identified that HIF-1-induced signaling in irradiated murine tumors follows the pattern of IR-induced reoxygenation and primarily occurs in the tissue with relatively high oxygenation levels. The authors linked this surprising finding to (1) ROS-mediated HIF-1α stabilization and (2) an increase in the translation of HIF-1-regulated transcripts following depolymerization of “stress granules” due to reoxygenation. Several studies suggested that ROS lead to HIF-1α stabilization, probably by reducing the activity of PHD enzymes [95–99]. However, other hypotheses have emerged, for example attributing ROS-mediated regulation of HIF-1α to the ROS-induced activation of the PI3K/AKT/mTOR and Ras/Raf/MEK/ERK pathways, which in turn leads to an increase in HIF-1α expression (see below). This intriguing topic is the focus of several recent reviews (see [100–103] and references therein). In addition to ROS, heat shock protein 90 (Hsp90) has also been implicated in HIF-1α protein stabilization following irradiation [104, 105].

The PI3K/AKT/mTOR and the Ras/Raf/MEK/ERK (also known as MAPK/ERK) signaling pathways are the major IR-responsive signal transduction cascades linking IR and HIF-1. Mutations in different entities of these pathways are considered key contributors to the hallmarks of cancer, most notably to malignant cell growth, survival, proliferation and metabolism [106, 107]. The HER family of receptor tyrosine kinases (RTKs), which includes the epidermal growth factor receptor (EGFR or HER1), are upstream of both of these pathways. An increase in their phosphorylation and thus activation status in response to IR activate these signaling cascades even in absence of the corresponding growth factor ligands [108, 109]. Furthermore, rapid, IR-induced activation of the Ras/Raf/MEK/ERK pathway has been linked to several other kinases, namely the extracellular signal-regulated kinase (ERK), c-Jun N-terminal kinase (JNK) and p38 mitogen-activated protein kinase (MAPK) [108, 110]. Interestingly, an increase in glucose availability has also been proposed as facilitator of IR-induced activation of the PI3K/AKT/mTOR pathway [111]. The activation of the PI3K/AKT/mTOR and Ras/Raf/MEK/ERK pathways in response to IR, in turn, leads to an increase in the expression of HIF-1α [111, 112].

##### Radiotherapy and the Hypoxic Tumor Microenviroment

Radiotherapy influences the immune cell compartment and the stroma of the tumor microenvironment (TME) [113–115]. The topic of the multifaceted role of radiotherapy at the interface of hypoxia-mediated immunosuppression and the anti-tumor immune response is comprehensively reviewed elsewhere [21, 115–122]. Effects of IR on the stroma can be divided into two topics: (1) the interaction with endothelial cells and the tumor vasculature and (2) the interaction with myofibroblasts and radiation fibrosis.

In response to IR, endothelial cells mount a stress response, followed by either recovery, or loss of function and cell death. The ultimate fate depends on multiple variables: the total dose, fractionation schedule, and the intrinsic TME properties [123]. The affected endothelial cells and vasculature lose their ability to deliver oxygen and nutrients to the tumor cells, leading either to an adaptive increase in hypoxia-mediated signaling or to indirect radiation-induced cell death (see also “Tumor hypoxia in the age of hypofractionation” below). Vascular endothelial growth factor (VEGF) is at the center of this interaction, as HIF-1 target gene and as essential mediator of tumor (re)vascularization [124]. VEGF activates the endothelial cells and promotes the formation of new blood vessels, thereby facilitating tumor cell survival and radioresistance. Apart from hypoxia-mediated signaling, the damage to the endothelial cells directly facilitates an inflammatory response, mediated in large part by nuclear factor kappa B (NF-κB) [125, 126]. This contributes to a radiation-induced inflammatory state, which in turn activates signaling pathways upstream of HIF-1 (as described above) [127].

Radiation-induced fibrosis (RIF) is a dose-limiting late side effect of radiotherapy. The development of RIF corresponds to an abnormal wound healing process in response to radiation injury, resulting in a self-propagating fibroproliferative state [113]. The process involves an early inflammatory response to IR-induced DNA damage, followed by endothelial cell dysfunction and hypoxia. Together, this leads to an abnormal activation of fibroblasts (called myofibroblasts in the activated state) and excessive deposition of collagen and other extracellular matrix proteins. Transforming growth factor beta (TGF-β) plays a central role in this process, with ROS-mediated posttranslational activation of TGF-β as one of the main initiating processes of RIF [128]. In addition to the rapid ROS-mediated activation, the production of TFG-β and several other profibrotic proteins such as connective tissue growth factor (CTGF) and platelet-derived growth factor (PDGF) is increased in response to hypoxia [129, 130]. Taken together, this illustrates the complex interaction between radiotherapy, hypoxia and fibrosis in the normal tissue, which is ultimately dose-limiting and thus contributes to radiotherapy treatment failure. Apart from their role in the normal tissue, fibroblast abnormalities, specifically identified in cancer-associated fibroblasts (CAFs) in the TME, may also directly contribute to cancer radioresistance [131]. The role of CAFs in radiotherapy has recently been reviewed elsewhere [132]. In the context of hypoxia, it is important to consider that - similarly to normal tissue - IR-induced, hypoxia-mediated signaling contributes to abnormal activation of CAFs. In turn, irradiated CAFs may facilitate various pro-tumorigenic processes, many of which are already directly activated by hypoxia. These include for example TGF-β-mediated epithelial-to-mesenchymal transition (EMT) [133], or the paracrine activation of pro-survival pathways such as the PI3K/AKT/mTOR- (through e.g. insulin-like growth factor 1 – IGF-1 [134]) and the Ras/Raf/MEK/ERK-pathways (through e.g. CXCL1 [135]). As described above, these pathways can in turn activate HIF-1, thus closing a vicious tumor-promoting circle.

##### Metabolic Reprogramming

Reprogramming energy metabolism is one of the hallmarks of cancer [106], which encompasses cancer-associated alterations in metabolic activities, supporting the altered metabolic demands of cancer cells and for tumor growth [136]. The Warburg effect or aerobic glycolysis is central to metabolic reprogramming and comprises a tumor cell-specific shift towards glycolysis and lactate production even under normoxic conditions. Even though aerobic glycolysis to lactate is less efficient than mitochondrial oxidative phosphorylation in terms of ATP generation, a high rate of glucose uptake provides sufficient precursors for anabolic pathways such as the hexosamine pathway, pentose-phosphate pathway (PPP) and one-carbon metabolism. Furthermore the mitochondrial tricarboxylic cycle is still fueled with a few percentages of pyruvate to generate intermediates now used as precursors e.g. for lipid synthesis, thus ultimately also supporting cell growth and proliferation [136–138].

As a transcriptional activator of glucose transporters (most importantly, GLUT-1) [139] and virtually all glycolytic enzymes, including lactate dehydrogenase A (LDH-A) and others, HIF-1 is closely interconnected with this metabolic network [40]. In addition to the upregulation of glycolytic enzymes, HIF-1 supports aerobic glycolysis by suppressing mitochondrial respiration, inhibition of mitochondrial biogenesis and induction of mitochondrial autophagy [41, 140, 141]. Finally, as described above, HIF-1 promotes tumor angiogenesis, which increases the availability of glucose and other nutrients. Overall, HIF-1 activity drives a strong glycolytic flux, which - as part of a positive feedback loop - keeps HIF-1 activity through pyruvate-mediated inhibition of α-ketoglutarate (α-KG) high [142].. In the absence of the co-factor α-KG, PHDs cannot hydroxylate HIF-1α, thus leading to its stabilization. Similar mechanisms link mutations in the metabolic enzymes isocitrate dehydrogenase 1 and 2 (IDH-1/2), fumarate hydratase (FH) and succinate dehydrogenase (SDH) to a stabilization of HIF-1 even under normoxic conditions: dysfunction of these three enzymes causes accumulation of D-2-hydroxyglutarate (D2HG), fumarate and succinate, respectively, which in turn interfere with PHD activity [136].

In the context of radiotherapy, glycolysis-induced activation of the PPP is one of the best examples illustrating how HIF-1-mediated metabolic reprogramming may directly confer radioresistance. Activation of PPP results in the regeneration of nicotinamide adenine dinucleotide phosphate (NADPH), which in turn reduces oxidized glutathione to protect cancer cells from ROS [136, 143, 144].

Furthermore, multiple positive feedback loops exist between aerobic glycolysis and radiotherapy-regulated signaling towards HIF-1 activity. The PI3K/AKT/mTOR pathway is one of the key drivers of aerobic glycolysis [145]. Harada et al. observed that IR-induced tumor reoxygenation increases glucose availability, also leading to the activation of the nutrient-sensing PI3K/AKT/mTOR pathway. The subsequent increase of HIF-1α activity further supported glycolysis [111]. However, HIF-1 is known to suppress PI3K/AKT/mTOR signaling in response to hypoxia and thus might also create a negative feedback loop [69, 146, 147]. Thus, in the context of hypoxia and radiotherapy, the role of the PI3K/AKT/mTOR pathway is multifaceted.

These examples only serve to illustrate the network complexity at the interface of tumor metabolism, hypoxia and radiotherapy. Many other signaling pathways, oncogenes and tumor suppressors are also closely connected to the radiotherapy response and are also linked to aerobic glycolysis. Originally identified as the major driver of RT-induced apoptosis in tumor cells, the tumor suppressor protein p53 is also activated under hypoxia in a HIF-dependent manner and thereby selects for a more aggressive treatment resistant tumor phenotype [46, 148–150]. More recent data, however, suggest an anti-apoptotic and p53-suppressing role for HIF-1 [151]. Interestingly, p53 also antagonistically regulates glycolysis and oxidative phosphorylation via transcriptional regulation of the downstream genes TP53-induced glycolysis regulator (TIGAR) and assembly of cytochrome c oxidase (SCO2). For further information, the reader is referred to [26, 143, 144, 152–155].

### The Significance of Hypoxia in Radiation Oncology

#### Detecting Tumor Hypoxia on the Clinical Level

The development of a reliable and routinely usable technique to determine the oxygenation status of human tumors has proven to be challenging. Considering the anatomical position of solid tumors, the range of oxygen tension in human tumors and the spatiotemporal heterogeneity of tumor hypoxia, such a technique would ideally be non-invasive and repeatable, highly sensitive, and with a high spatial and temporal resolution.

Due to technological limitations, early attempts to determine the oxygenation status of human tumor had to rely on indirect measurements. These primarily focused on the vascular component of the tumor, correlating hypoxia to e.g. vascular density based on immunohistochemistry or the perfusion status based on computed tomography (CT), positron emission tomography (PET) or magnetic resonance imaging (MRI) [32, 156]. While informative, such methods were not able to comprehensively capture the complexity of tumor oxygenation, that does not only depend on isolated characteristics of the vascular component.

The introduction of the “Eppendorf” polarographic needle electrode in the 1990s offered for the first time the possibility to measure the oxygenation status of human tumors directly and reproducibly, albeit invasively and with a low spatial resolution [157]. Although these disadvantages precluded its introduction into routine clinical practice, the “Eppendorf” electrode remains historically as one of the major contributors to the recognition of the importance of tumor hypoxia on the clinical level.

In the following years, numerous other techniques for hypoxia detection were developed and refined. In general, the two main approaches include: (1) *ex vivo* techniques such as immunohistochemistry or gene expression analysis performed on invasively obtained patient material and (2) non-invasive or minimally invasive imaging techniques, which enable *in vivo* hypoxia detection [32, 50, 158–161]. Immunohistochemistry is an affordable approach, which allows detection of hypoxic cells with high spatial resolution. Targets visualized by immunohistochemistry include endogenous, hypoxia-induced markers (e.g. HIF-1α, carbonic anhydrase IX - CAIX, HIF-1α, glucose transporters GLUT-1 and GLUT-3, osteopontin – OPN) and exogenous markers that specifically accumulate in hypoxic regions (e.g. 2-nitroimidazole - pimonidazole). As a major advantage, endogenous markers do not require planned intravenous administration of a chemical moiety prior to biopsy, thereby enabling also studies on archival material. On the other hand expression of the majority of endogenous markers is regulated by a multitude of factors other than hypoxia, thereby limiting the specificity of this approach [162]. Furthermore, the pO_2_ level at which a hypoxia-induced increase in gene expression occurs depends on the endogenous marker of choice but does not necessarily correlate with the pO_2_ level most relevant to radiation oncology (see above).

The most widely used exogenous markers, 2-nitroimidazoles, bind to their targets specifically at pO_2_ levels below 10 mmHg [161]. The accumulation of these compounds in respective tumor areas therefore highly correlates with areas of “radiobiological hypoxia”. An additional advantage of exogenous markers is the possibility to label them with probes detectable by non-invasive imaging methods, such as single photon emission computed tomography (SPECT), PET or MRI (see below). Aside from immunohistochemistry, gene expression profiling has recently been established as a promising tool for the *ex vivo* classification of hypoxic tumors [163]. This molecular biology technique assesses the transcriptional response of the tumor to its microenvironment by using RNA sequencing to detect hypoxia-specific gene upregulation on the whole-tissue as well as on the single-cell level. The selection of relevant genes can be tailored to specific tumors and even individual patients. The resulting “hypoxia gene signature” can then be used to stratify tumors as normoxic or hypoxic, and to guide the therapy in line with personalized hypoxia-oriented treatment approaches.

Imaging techniques are non-invasive or minimally invasive approaches that mostly rely on the administration of exogenous hypoxia-specific compounds bound to probes that can be detected by various imaging modalities. Specialized MRI approaches such as blood oxygen level dependent (BOLD) imaging, however, can indirectly quantify tumor hypoxia without a chemical probe. In addition to their non-invasiveness, imaging techniques are generally sensitive and repeatable. Furthermore, it is possible to obtain multiple measurements over time, thereby capturing at least partially the temporal fluctuation of tumor hypoxia, depending on the temporal resolution of the technique. Despite these advantages, imaging methods still face many limitations, mainly stemming from the spatial and temporal resolution, affordability and availability of such systems. Nevertheless, of the various techniques researched to date, PET is the most widely used in the clinical setting; PET hypoxia tracers such as 18F-fluoromisonidazole (FMISO) and 18F-fluoroazomycin arabinoside (FAZA) have been applied in a number of clinical studies [158, 159].

#### Recognition of the Role of Tumor Hypoxia in Radiation Oncology

While the evidence from early preclinical studies strongly suggested that tumor hypoxia and consequently its modification could be highly relevant for the radiotherapy response, initial attempts at implementation of oxygen-mimicking drugs in the treatment protocol in the 1970s failed to demonstrate a significant improvement over radiotherapy alone [164]. In addition to this initial lack of positive results, reliable *in vivo* detection and quantification of hypoxia was a major hurdle at the time (see above). The introduction of the “Eppendorf” electrode, as a robust and reproducible method to measure the oxygenation status of human tumors, paved the way towards the first irrefutable evidence of hypoxia-induced radioresistance. Hoeckel et al. [165] and Fyles and al [166]. demonstrated the predictive power of tumor oxygenation in the radiation response of cervical cancer, while Nordsmark et al. provided first strong clinical evidence of the importance of tumor hypoxia in the radioresistance of head and neck tumors [167]. Controlled clinical trials employing modification of tumor hypoxia undeniably demonstrated the significance of hypoxia for the treatment response to radiotherapy, in particular for squamous cell carcinoma of the head and neck [168, 169].

#### The Relevance of Hypoxia in Conventional Radiotherapy

**Fig. 2:**
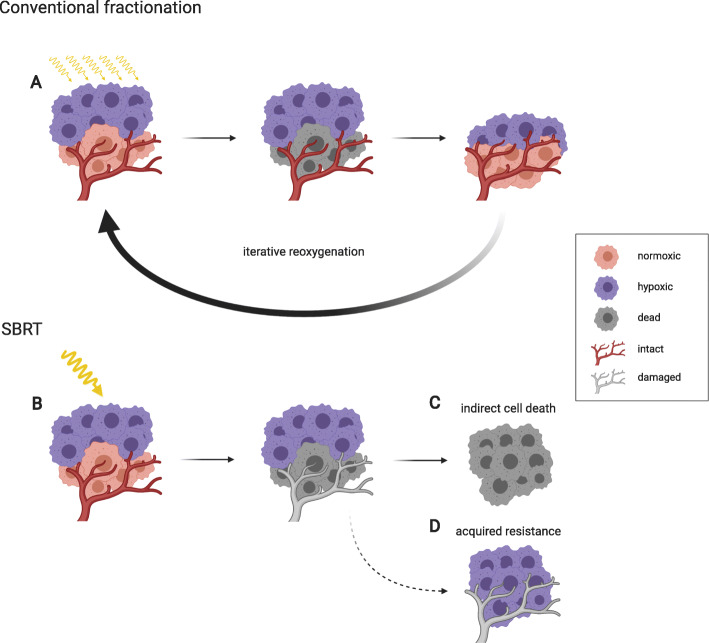
The relevance of hypoxia for conventional fractionated RT and SBRT. In conventional low dose fractionated radiotherapy, the hurdle of tumor hypoxia is overcome by iterative reoxygenation of radiation-resistant hypoxic tumor cells after irradiation and cell killing of oxygenated radiation-sensitive tumor cells (**a**). In SBRT (**b**), single high doses of ionizing radiation will effectively kill tumor cells and damage the tumor vasculature, leading to secondary tumor cell death (**c**) or will induce hypoxia-related resistance mechanisms (**d**). Radiotherapy-induced toxicities in co-irradiated normal tissues often limit the required dose escalation for both low dose fractionated RT and SBRT to achieve tumor control

Conventional fractionation has been the mainstay of radiation oncology for decades. By dividing the total radiation dose into 1.8 to 2 Gy fractions given five days per week up to a total dose of 40–70 Gy, conventional fractionation exploits the biological differences between the tumor and the normal tissue, resulting typically in less damage of the normal tissue for the same level of tumor control. This increase in the therapeutic ratio is ultimately the result of a complex interplay between different factors, summarized by Withers as the four R’s of radiotherapy: repair of sublethal cellular damage, repopulation of cells following radiation, redistribution of cells within the cell cycle and reoxygenation of the surviving cells [32, 170, 171]. The phenomenon of reoxygenation occurs after depletion of radiosensitive normoxic cells in response to a single low dose fraction of IR (Fig. 2a). Immediately after IR gradual reoxygenation of the more hypoxic surviving tumor cells occurs within hours or days. With conventional fractionation, this iterative process of reoxygenation is taking place continuously during the course of treatment, thereby partially improving tumor control without an influence on the normoxic normal tissue [32]. Notably, given the proven negative influence of hypoxia on the success of conventional radiotherapy as mentioned above, reoxygenation on its own is evidently insufficient to fully overcome hypoxia-induced radioresistance.

#### Tumor Hypoxia in the Age of Hypofractionation

In contrast to conventional low dose fractionated regimens, hypofractionation describes a radiation treatment where the total dose is given in a smaller number of larger (> 2 Gy) fractions, resulting in an overall shorter treatment time. When given as a single dose or a small number of fractions, usually with the dose of 8–30 Gy per fraction, the treatment is called stereotactic body radiotherapy (SBRT) [172]. The interest in hypofractionation has been rising steadily along with the technological advances in image guidance and highly conformal dose delivery, which make it possible to deliver a large dose to the tumor, while keeping the dose to the surrounding normal tissues acceptable. Today, hypofractionation is considered routine clinical practice in the treatment of certain disease sites, such as the breast, lung, liver and prostate [173]. Radiobiological implications of hypofractionation, including possible additional biological effects, have been reviewed elsewhere [174, 175]. In short, the success of hypofractionation seems to largely be attributable to an increase in the biologically effective dose to the tumor cells, although vascular damage and antitumor immunity stimulation at high doses per fraction might also play an important role. The question of the relevance of hypoxia for hypofractionation similarly remains open (Fig. 2b). On one hand, hypoxia was suggested to be more detrimental for hypofractionated treatments, primarily due to the loss of reoxygenation and possibly due to vascular damage, leading to oxygen deprivation [174, 176]. On the other hand, high-dose-mediated vascular damage was demonstrated to induce secondary cell death in hypoxic cells, which would normally escape direct IR-mediated death; such an effect might counteract the potential loss of reoxygenation and explain the success of hypofractionation shown in clinical trials [175]. In a recent study investigating for the first time tumor hypoxia in SBRT on the clinical level [175], Kelada et al. demonstrated that high single doses of radiation given to lung cancer patients as a part of SBRT may induce an increased and persistent state of hypoxia. Overall, the significance of tumor hypoxia in the age of hypofractionation therapy remains elusive, and future clinical investigations are necessary. At the same time the shift from classic fractionated radiotherapy regimens to SBRT resulted in a new search for hypoxia-modifying agents to be applied as part of a combined treatment modality with radiotherapy.

### Targeting Tumor Hypoxia

**Fig. 3:**
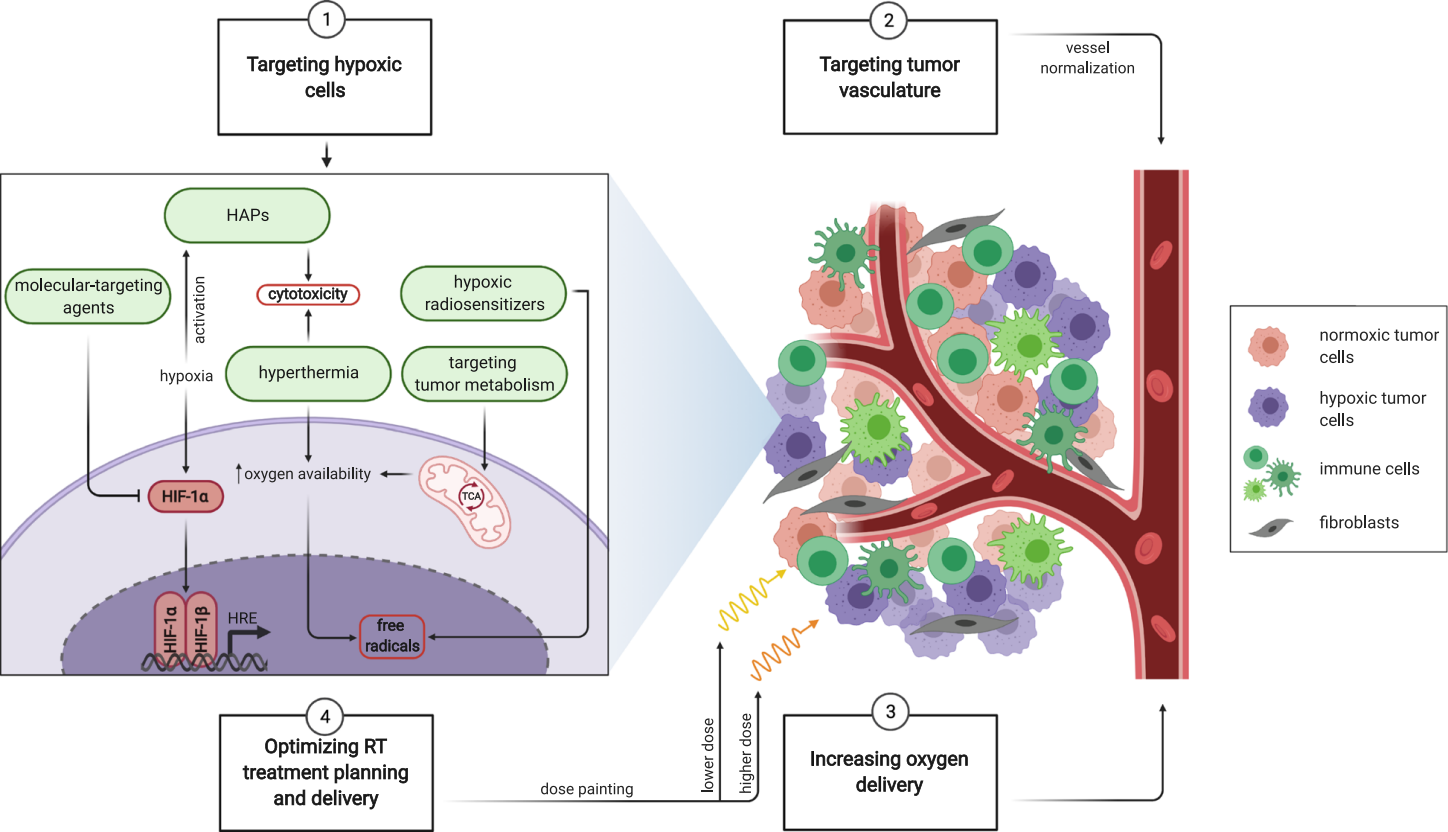
Different approaches to targeting tumor hypoxia. Several, in part complementary pharmaceutical and radiotherapeutical approaches have been developed since the identification of hypoxia as major resistance factor for successful radiotherapy, including pharmaceutical agents that directly target hypoxic tumor cells and the tumor vasculature, and strategies that modify oxygen delivery to hypoxic tumor areas. In addition to iterative reoxygenation as part of low dose fractionated radiotherapy regimes, improved treatment planning coupled with hypoxia-specific imaging results in novel radiotherapy treatment delivery with highest dose conformity. *HAPs* hypoxia-activated prodrugs, *HRE* hypoxia response element, *TCA* tricarboxylic acid cycle

Several strategies have been developed during the last decades to overcome the hurdle of tumor hypoxia for successful radiotherapy. These strategies can be grouped into fundamentally different approaches (Fig. 3). While hypoxic radiosensitizers, hypoxia-activated prodrugs and molecular-targeting agents are preferentially effective in hypoxic parts of the tumor, other approaches aim to increase oxygen availability in the tumor and subsequently to reduce tumor hypoxia and radiation resistance. Finally, radiotherapy-based approaches rely on the concept of personalized medicine to specifically target tumor hypoxia and thereby optimize the treatment planning and delivery on an individual level.

#### Targeting Hypoxic Cells

##### Hypoxic Radiosensitizers

The prototype of hypoxic radiosensitizers are the electron-affinic nitroimidazoles, such as misonidazole, etanidazole, pimonidazole and the clinically approved nimorazole, which is given as standard of care to patients for head neck cancer radiotherapy, in particular in Denmark [169]. Originally, already more than 50 years ago, nitrobenzenes followed by the nitroimidazoles were proposed to act as oxygen mimetic agents and to react in hypoxic cells with short-lived, oxidizing, IR-induced free DNA radicals to generate cytotoxic DNA strand breaks, thus creating an adjuvant therapeutic effect [177]. Notably, while oxygen is metabolized already by normoxic cells close to the microvessel, hypoxic radiosensitizers could diffuse further to reach also hypoxic zones within the tumor.

Unfortunately, and despite the clarity of the concept, several reasons might have contributed that these hypoxic radiosensitizers did not find their recognition in the clinical routine. While they indeed enhanced the radiation response of hypoxic tumor cells *in vitro* and of tumor xenografts *in vivo* in response to large single doses, hypoxic radiosensitizers did not prove as effective in response to a conventionally fractionated, low dose treatment regimen [164, 178, 179]. Subsequently, these compounds failed to significantly improve radiation therapy as part of classic fractionated low dose treatment regimens with concomitant reoxygenation, rendering them less potent. Furthermore, due to severe toxicities, especially neurotoxicity, many of these compounds could not be applied in sufficiently high doses. For these reasons, there is an ongoing effort required to identify novel drugs and/or strategies that would have lower systemic toxicity but retain hypoxic radiosensitization. One of such novel compounds is RRx-001, a nitric-oxide-generating dinitroazetidine radiosensitizer with direct cytotoxic effects [180, 181]. Based on promising preclinical data, RRx-001 is now being tested as part of a combined treatment modality together with radiotherapy in the treatment of primary brain tumors (NCT02871843) and brain metastases (NCT02215512, [182]).

Lack of patient selection is an additional reason for the failure of hypoxic radiosensitizers on the clinical level. Using a 15-gene hypoxia classifier to detect a hypoxia gene signature (see above) [183], Toustrup et al. retrospectively analyzed the beneficial effect of nimorazole in head and neck squamous cell carcinoma (HNSCC) patients treated as part of the Danish Head and Neck Cancer Study (DAHANCA) 5 [184–186]. While the original study indeed found a beneficial effect from the addition of nimorazole without pretreatment classification (which eventually led to the introduction of nimorazole into the clinical use), the gene-signature-based classification showed a markedly increased effect of nimorazole in patients whose tumors were classified as more-hypoxic. These findings were confirmed in the IAEA-HypoX study (NCT01507467), where the retrospective analysis of HNSCC patients treated with accelerated radiotherapy in combination with nimorazole demonstrated an increased benefit of nimorazole addition in more-hypoxic tumors [187]. Patient classification based on the hypoxic status of the tumor before treatment administration could therefore uncover previously undetected treatment benefits in the “more-hypoxic” subset of patients. Consistently, prospective stratification based on the hypoxic status of the tumor is now being evaluated as part of two HNSCC clinical studies investigating the addition of nimorazole: gene-signature-based classification is included in the non-inferiority DAHANCA 30 study (NCT02661152) that aims to verify the hypothesis that there is no benefit in nimorazole addition to less-hypoxic patients; FAZA-PET-based classification is being evaluated a part of the DAHANCA 33 study (NCT02976051).

In addition to the ongoing efforts to optimize the use of hypoxic radiosensitizers and to identify novel compounds, it could be of interest to see a renaissance of former drugs, e.g. the hypoxia-activated prodrug tirapazamine (see below), and to probe them as part of hypoxia-stratified, personalized trials with SBRT. Despite the recent success of SBRT, hypoxic tumor entities still represent a major challenge for a hypofractionated treatment regimen with only a few high dose fractions of IR.

##### Hypoxia-Activated Prodrugs

Hypoxic radiosensitizers, such as nitroimidazoles, are biochemically reduced under low pO_2_ conditions to become cellular cytotoxins and are therefore already more toxic to hypoxic than to normoxic cells independent of IR. These findings paved the way for the hypoxia-selective generation of bioreductive prodrugs, which are activated by enzymatic reduction in hypoxic tissues [188]. Five different chemical moieties have been exploited as prodrugs for bioreduction under hypoxic conditions. These include nitro-groups (e.g. nitroimidazoles), quinones (e.g. mitomycin C), aromatic N-oxides (e.g. tirapazamine (TPZ)), aliphatic N-oxides (e.g. AQ4N) and transition metals (e.g. cobalt (III) and copper (II) complexes to release cytotoxic agents ligands). These prodrugs are reduced by concerted mechanisms, which involve either one-electron reductases followed by fragmentation or further reduction of the initially formed prodrug radicals; or two-electron reductions of specific prodrugs. In normoxic conditions, the initial reaction is reversible, while under hypoxic conditions these prodrugs become stable cytotoxins, and act as mono−/di-functional DNA alkylators, intra- and interstrand crosslinkers, and DNA-strandbreakers or additionally poison specific enzymes involved in correct DNA-replication and repair, such as topoisomerase II by TPZ [5, 189].

Even though some of these hypoxia-activated prodrugs (HAPs) were also shown to physico-chemically sensitize for a combined treatment modality with IR (synergistic effect), HAPs rather biologically cooperate with radiotherapy, which is less potent in the hypoxic tumor environment (additive effect). Many of these HAPs have been tested on the preclinical and clinical levels but often with only minimal success in the clinics. Besides differences with regard to classic pharmacokinetic aspects, such as stability and diffusability, the different classes of HAP also vary in their oxygen-dependence for activation, which affects normal tissue toxicity. HAPs that are activated at intermediate rather than very low oxygen tension in the tumor (i.e. at the level of “radiobiological hypoxia” as describe above), such as TPZ and its derivatives, might be most potent to eliminate the relevant fraction of tumor cells at this critical level relevant for radiation resistance. However, such agents run the inherent risk of prodrug activation in normal tissues, which can reach these intermediate oxygen tension values in physiological conditions, thereby resulting in concomitant, limiting side toxicities. Indeed, the initial clinical studies of TPZ in combination with cisplatin and/or radiotherapy failed to demonstrate an additional benefit within the tolerable dose range [190]. However, retrospective analyses later suggested a lack of patient stratification and issues with radiation delivery as potential culprits for this failure [190–192]. Three clinical studies are still ongoing, all of them investigating the combination of TPZ and chemo- and immunotherapy in hepatocellular cancer (NCT03259867, NCT02174549, NCT03145558). A combination of TPZ with radiotherapy is currently not being investigated on the clinical level.

Dose-limiting toxicities represent a major hurdle for hypoxia-activated prodrugs. Therefore, a strong interest in the development of novel, less toxic compounds exist. These include new derivatives of TPZ (for example SN30000, a TPZ analogue that demonstrated superior activity in preclinical studies [193, 194]) or compounds with chemical moieties (nitro-compounds and quinones) that are only activated under severe hypoxia.

Activated prodrugs could also induce a bystander effect by the diffusion of the active cytotoxic agents from areas of severe hypoxia to tumor areas with intermediate levels of tumor hypoxia and normoxia. The 2-nitroimidazole conjugated bromoisophosphoramide TH-302 (evofosfamide) and dinitrobenzamide PR-104 represent prototypes of such HAPs [195–197]. TH-302 releases upon one-electron reduction and fragmentation a diffusible nitrogen mustard derivative with cytotoxic activity [198]. Although a bystander effect through diffusion was initially thought to substantially contribute to its single-agent activity, recent data suggest the absence of such an effect [199, 200]. Furthermore, despite the promising preclinical data and phase I/II clinical trials [201, 202], TH-302 failed to meet the primary endpoint in the two phase III clinical studies (NCT01440088, NCT01746979). Notably, despite the preclinical evidence of benefit in combination with radiotherapy [195, 203–205], all trials except one (NCT02598687 failed to meet the primary endpoint) only combined chemotherapy with TH-302. Currently, no clinical trials with TH-302 are open. Nitrogen mustard-DNA crosslinking-based PR-104 is another novel hypoxia-activated prodrug that failed to fulfill the expectations derived from preclinical data; dose-limiting hematological toxicities prevented the advancement of the drug beyond phase I/II clinical trials [196, 206–209]. Novel nitrobenzamides based on PR-104 are being developed with CP-506 showing interesting results on the preclinical level [210]. One of the major questions to be resolved is the relevance of scheduling of the combined treatment modality of hypoxia-activated prodrugs in combination with high-dose radiotherapy [203]. Furthermore, adjuvant integration of immune response regulatory agents as part of a combined treatment modality might be a promising approach. Following treatment with HAPs the tumor microenvironment might be less immune suppressive [211].

Of interest are not only prodrugs that release DNA-oriented cytotoxins but also bioreductive prodrugs which release diffusible inhibitors of non-genotoxic molecular targets. For example, PR-610 releases an irreversible inhibitor of the human EGFR upon bioreductive activation [212]; the phase I clinical trial NCT016312790 had to be terminated due to unacceptable toxicities. TH-4000 (tarloxotinib) is another prodrug of an EGFR inhibitor that advanced to phase II clinical trials as a single agent. The initial two trials in non-small cell lung cancer (NSCLC) and HNSCC [213, 214] did not meet the interim milestone and were terminated, while the RAIN study (NCT03805841) investigating TH-4000 in NSCLC and other advanced solid tumors is ongoing.

The use of such novel bioreductive prodrugs alone or in combination with IR could increase the local concentration of the active agent in the tumor and could lead to a more personalized and even synergistic treatment approach. Future, intense preclinical and clinical investigations will have to demonstrate under which circumstances a (neo-)adjuvant or a concomitant treatment regimen of these bioreductive cytotoxins with (hypo)fractionated radiotherapy will be most effective.

##### Molecular-Targeting Agents

The identification of key molecular mechanism orchestrating the cellular response to hypoxia uncovered new potential targets for overcoming hypoxia-induced treatment resistance. Targeting the HIF-1 pathway is a particularly interesting approach, as it is a key modulator of the hypoxia-induced phenotype and simultaneously regulates a plethora of resistance-inducing mechanisms (see above). Thus, HIF-1 targeting agents can reverse an aggressive phenotype on their own and at the same time synergize with radiotherapy. The HIF-1 signaling network can theoretically be targeted on multiple levels: by targeting the upstream pathways that regulate HIF-1 expression, by inhibiting HIF-1 protein expression, stability and function and by interference with downstream targets of HIF-1.

The two pathways that are most commonly implicated as upstream regulators of HIF-1 expression are the PI3K/AKT/mTOR and Ras/Raf/MEK/ERK pathways [111, 112]. These are ubiquitous and intensively investigated signaling pathways with pleiotropic effects. Subsequently, interference with these pathways does not specifically inhibit HIF-1, but rather results in a multitude of complex and interdependent effects. As such, the topic of targeting of these pathways in combination with radiotherapy is beyond the scope of this paper. Similarly, HIF-1 modulates the expression of hundreds of genes, and as such inhibition of downstream HIF-1 targets is only partially covered in this review. For a more comprehensive overview, the reader is referred to e.g. [26, 215, 216].

Both rational designed approaches, e.g. with RNA antagonists, and high-throughput screenings, e.g. with chemical libraries, were used to identify HIF-signaling interfering agents and thus, the landscape of HIF-1 inhibitors currently comprises a multitude of compounds with pleiotropic mechanisms. Multiple drugs such as EZN-2968, PX-478, topotecan, bortezomib, vorinostat, ganetespib (STA-9090) and geldanamycin have been reported to (indirectly) target HIF-1 protein expression or stability. EZN-2968 is a HIF-1α mRNA antagonist with evidence of preclinical activity [217] and encouraging proof-of-concept data from a phase I trial in patients with advanced solid tumors [218] and awaiting results from two insofar unpublished phase I studies (NCT00466583, NCT02564614). The small molecular compound PX-478 has been reported to act on multiple levels, inhibiting transcription, translation and deubiquitination of HIF-1α [219, 220]. In combination with radiotherapy in prostate cancer [221] and with gemcitabine in pancreatic cancer [222], PX-478 showed encouraging results on the preclinical level. The initial findings from the phase I clinical trial in patients with advanced solid tumors (NCT00522652) demonstrated acceptable tolerance in the dose range achieving detectable HIF-1 inhibition [223]. Topotecan is a known chemotherapeutic agent that inhibits topoisomerase I. Surprisingly, a high-throughput screening for HIF-1 inhibitors identified topoisomerase (Topo)-I inhibitors to repress HIF-1 transcriptional activity [224]. A pilot clinical trial confirmed the anti-HIF-1 activity in patients with advanced solid tumors [225]. Topotecan is a part of numerous ongoing clinical trials, however the majority does not investigate its role in the context of hypoxia. Bortezomib is a proteasome inhibitor, clinically used in the treatment of multiple myeloma. Besides its primary mechanism for protein stabilization, recent studies suggest that bortezomib also interferes, presumably as secondary effect, by inhibiting upstream regulators of HIF-1α [226], repressing HIF-1α translation [227] or by blocking HIF-1-mediated transcription [228, 229]. The radiosensitization effect of bortezomib in hypoxic cervical cancer cells [230] and esophageal squamous cancer cells [231] has been attributed specifically to HIF-1 inhibition, while other studies suggested different mechanism to dominate the increase in radiosensitivity [232–235]. The histone acetylase inhibitor vorinostat interferes with HIF-1 signaling through translation inhibition, increase in ubiquitination and modulation of nuclear translocation [236, 237]. Preclinical data support a radiosensitizing role of vorinostat through multiple different mechanisms [238–240], including in the setting of hypoxia [241, 242]. Ganetespib, geldanamycin and its analogues tanespimycin (17-AAG) and alvespimycin (17-DMAG) all inhibit Hsp90, which in turn leads to enhanced degradation of HIF-1α [105, 243–246]. Considering the pleiotropic effects of Hsp90 inhibition, these compounds have been extensively tested also in combination with radiotherapy, however not yet in the context of hypoxia.

Inhibitors of HIF-1 transcriptional activity, including for example echinomycin, PX-12 and chetomin, comprise a class of molecular hypoxia-targeting agents with potentially much higher specificity than the compounds mentioned above. Overall, this class of compounds is at an earlier stage of development compared to the aforementioned HIF-1 inhibitors, with only limited testing as part of a combined treatment modality with IR. Echinomycin is a DNA intercalator for which the original clinical trials in the early 1990s failed to demonstrate an anti-tumor effect in an acceptable dose range [247]. Despite these setbacks, preclinical research on echinomycin continued, and in 2005 echinomycin was revealed to act as a potent inhibitor of HIF-1 DNA-binding [248]. In parallel, to overcome toxicities, novel derivatives and formulations of echinomycin are being developed, such as the YK-2000 and liposome-encapsulated echinomycin [247, 249]. There are currently no clinical trials investigating echinomycin or its derivatives. PX-12 is an inhibitor of thioredoxin-1 with multiple proposed mechanisms of cytotoxicity, of which HIF-1 inhibition and ROS generation are of particular interest in the context of hypoxia and radiotherapy [250–253]. PX-12 has so far only been clinically tested as a single agent, with the lack of an anti-tumor effect leading to the termination of a phase II study [254]. Chetomin is a natural product that inhibits HIF-1 transcriptional activity by blocking the interaction between HIF-1α and its transcriptional coactivator p300 [255]. In vitro data demonstrated a radiosensitization effect in hypoxic glioma, fibrosarcoma and osteosarcoma cells [256–258]. In a spontaneous lung cancer model and NSCLC xenografts, chetomin inhibited tumor growth without observable adverse effects [259].

A more detailed overview of HIF-1-targeting molecular agents, along with insights into emerging compounds can be found in [260, 261]. Overall, the landscape of HIF-1 inhibitors currently comprises a multitude of compounds with pleiotropic mechanisms. Given that HIF-1α proteins are very short-lived with a high turnover, it is not surprising that such compounds show seemingly specific effects on the HIF-1 system because they occur before other cellular effects become apparent. While these HIF-1-directed agents are indeed in various stages of preclinical and even clinical research in the context of hypoxia, it should be emphasized that to date, there are no compounds that could be convincingly considered as specific HIF-1-targeting molecular agents. The main mechanism of action towards increased cell killing in combination with radiotherapy similarly remains to be investigated. HIF-1 targeting could sensitize radioresistant cells to IR-induced cell death or it could simply target the aggressive hypoxia-induced phenotype.

HIF-2 as a target in combination with radiotherapy has been less investigated. Notably, HIF-2α is an emerging target in clear cell renal cell carcinoma, with the compound PT2385 as a first-in-class, highly specific HIF-2α inhibitor currently tested in clinical trials [262]. Several *in vitro* studies observed a correlation between increased HIF-2α levels with radioresistance and demonstrated an increase in radiosensitivity following HIF-2α inhibition [263–267]. However, the interaction of HIF-2α inhibitors with radiotherapy on the clinical level is currently difficult to predict.

##### Targeting Tumor Metabolism

As described above, tumor-specific metabolic reprogramming is an important contributor to radioresistance.

Between the different targeting strategies, increasing oxygen availability by targeting oxidative phosphorylation is emerging as a particularly interesting approach, with sufficient tumor and stroma cells still using oxidative phosphorylation as a major source for ATP generation under normoxic conditions [136, 268]. Therefore, compounds reprogramming those cells to anaerobic glycolysis or directly targeting mitochondrial respiration and metabolism sensitize for IR-induced cytotoxic DNA damage by creating (a window of) enhanced tumor oxygenation due to reduced oxygen consumption [269, 270]. A promising candidate belonging to this group of compounds is metformin, one of the most commonly prescribed drugs in the treatment of type II diabetes. The potential role of metformin in cancer development and treatment was first observed in retrospective studies of diabetic cancer patients [271]. Zannella et al. showed that this effect can, at least partially, be attributed to an improved tumor oxygenation and subsequent increase in radiosensitivity [272]. Mechanistically, this occurs through metformin-mediated inhibition of the mitochondrial complex I [273]. Inhibition of complex I, in turn, leads to a redistribution of oxygen and accumulation of α-KG, both of which activate PHDs, and subsequently results in the degradation of HIF-1α. Thus, the contribution of metformin to increased radiosensitivity can occur on two levels: (1) a direct increase in cytotoxic IR-induced ROS through an increase in oxygen availability and (2) an inhibition of HIF-1-mediated prosurvival- and radioresistance-inducing signals [274]. Following these intriguing preclinical observations, metformin also showed promising results in NSCLC patients receiving chemoradiotherapy (NCT02109549, [275]) and in colorectal cancer patients (NCT03053544). Currently, four clinical trials are investigating metformin as a radiosensitizing, tumor oxygenation-enhancing drug (NCT04170959, NCT03510390, NCT02394652, NCT04275713). The anti-malarial agent atovaquone is an additional example for an “old drug”, that was repurposed in an attempt to exploit its ability to inhibit the mitochondrial complex III [276]. The ability of atovaquone to modify tumor hypoxia and thereby improve the efficacy of radiotherapy was demonstrated in cell lines and tumor xenografts [277] and is currently being tested in NSCLC patients (NCT0262808). The third example of a repurposed drug is papaverine, a smooth muscle relaxant and complex I inhibitor whose radiosensitizing properties are being tested in a phase I clinical trial in combination with SBRT ([278], NCT03824327). There are numerous other examples of oxidative phosphorylation inhibitors that have shown impressive results in cell lines and mouse models, but have not yet been able to advance to the clinical level; these include arsenic trioxide [279, 280], nonsteroidal anti-inflammatory drugs [281] and glucocorticoids [282]. Finally, there is a multitude of promising additional emerging compounds such as BAY-87-2243 and IACS-010759. Both compounds demonstrated antitumor activity as single agents ([283–285], NCT03291938, NCT01297530) and in combination with radiotherapy [286] or radioimmunotherapy [287].

Apart from the inhibition of mitochondrial functions, other potential targets for radiosensitization in the network of tumor metabolism include for example α-KG as a rate-limiting substrate of the PHD family of enzymes [288], and a broad spectrum of enzymes involved in glycolysis and the PPP (reviewed in [26, 153, 289]). Overall, current mechanistic insights indeed support the role of numerous metabolic entities in the development of radioresistance, however the effects of interference with these entities as targets in the context of radiotherapy remains to be investigated.

##### Hyperthermia

Local application of heat in the range of 39–45 °C, i.e. hyperthermia, is a long-known anti-cancer treatment modality with proven radiosensitizing and cytotoxic effects that preferentially occur in the hypoxic regions (136–140). As part of a combined treatment with chemo- or radiotherapy, hyperthermia is already approved for routine clinical use in several countries. The radiosensitizing effect is thought to occur due to the improvement of oxygen delivery to the tissue [290, 291] and due to interference with the DNA damage response [292–298]. Cytotoxic effects can be direct and indirect: heat can directly induce necrosis, apoptosis and mitotic catastrophe [299–301] and with an applied temperature above 42 °C, heat will indirectly kill by inducing vascular damage [302]. At the same time, however, exposure to elevated temperatures might result in Hsp90-mediated HIF-1α protein stabilization even under normoxic conditions, thereby potentially counteracting the radiosensitizing effect [105]. The details and the relative importance of these effects remain elusive. The optimal temperature, duration and timing of heat exposure still need to be identified. Nevertheless, there is an active interest for hyperthermia in the radiation oncology community, illustrated by the fact that there are currently 16 active clinical trials investigating the combination of hyperthermia and radiotherapy. The addition of hypoxia-targeting compounds to the combination of radiotherapy and hyperthermia might be an interesting future approach, as demonstrated by Sadeghi et al. [303] who investigated temperature-sensitive liposomes loaded with the hypoxic radiosensitizer pimonidazole.

#### Targeting the Tumor Vasculature

In contrast to the aforementioned approaches, which primarily target hypoxic tumor cells, inhibitors of angiogenesis (IoAs) aim at cells of the tumor vasculature. Intuitively, IoAs should destroy the tumor vasculature and thus increase tumor hypoxia. Early preclinical data investigating the combination of radiotherapy with IoAs in mouse tumor models offered conflicting results: while there was evidence of an increase in radioresistance following an IoA-mediated increase in hypoxia, there were also reports of a delayed tumor growth when combining the two treatment modalities [304–307]. Specifically, the fumagalin-derivative TNP-470, which specifically inhibits endothelial cell proliferation, and the endogenous IoA angiostatin, were demonstrated in several animal tumor models to enhance tumor growth delay and to surprisingly and counterintuitively increase tumor oxygenation, when used in combination with IR [305, 306, 308, 309]. In our study using a murine mammary carcinoma allograft model, we found that an IoA-induced increase in tumor hypoxia is nullified when combined with irradiation, most probably due to the decreased demand for oxygen by the drastically reduced number of tumor cells surviving the first few fractions of irradiation [310]. Nevertheless, the existence of contradictory data and the potential to antagonize radiotherapy contributed for a long time to the reservations to combine IoAs with radiotherapy in the clinics.

In 2001, Jain coined the (now widely accepted) term “normalization” of the tumor vasculature to explain the paradoxical findings of IoA-induced potentiation of the effects of chemotherapy and radiotherapy [311–313]. Tumor hypoxia is caused by a dysregulated tumor vasculature and an overproduction of angiogenic growth factors. Anti-angiogenic strategies were demonstrated to destruct immature microvessels, to stabilize leaky vessels and to remodel the dysfunctional tumor vasculature to a normal phenotype with increased tumor blood flow and oxygen delivery. This process could contribute to the increased treatment response originally observed and now aimed for by the combined treatment modality of IR with IoAs. A major obstacle though is that normalization of the tumor vasculature is a transient process only occurring within a short time window and highly dependent on correct dosing of the respective inhibitor and on the individual tumor environment. Nevertheless, this line of thinking resulted in preclinical studies, that successfully demonstrated an enhanced radiation response when tumors were irradiated during a transient increase of tumor oxygenation [314–316]. These studies were performed on tumor xenografts and orthotopic tumor models treated with different classes of anti-angiogenic agents, such as the VEGF-directed antibody bevacizumab, the VEGF-receptor directed antibody DC101, the anti-angiogenic peptide anginex or multiple VEGF-RTK-inhibitors [309, 317–321]. These preclinical findings motivated a large number of clinical trials, the majority of which investigated the combination of bevacizumab with radiotherapy, but also the broad-sprectrum IoA endostatin, RTK-inhibitors sorafenib, sunitinib, vandetanib and semaxanib; and thalidomide (extensively reviewed in [314, 322]). Overall, the results of these studies so far have failed to show a substantial benefit of the addition of IoAs to a radiotherapy treatment regimen; but do not exclude the feasibility of such an approach, with patient selection and optimization of dosing and scheduling remaining the key challenge. Interestingly, not only VEGF but also other pro-angiogenic factors such as placental growth factor (PlGF) are secreted in response to irradiation and in a dose- and time-dependent manner. Our own recent studies showed a strong paracrine vasculature-protective role of PlGF as part of a p53-regulated IR-induced resistance mechanism and suggest PlGF as a promising target for a combined treatment modality with radiotherapy [323]. Furthermore, not only endothelial cell-directed compounds, but also tumor cell signaling-directed agents may contribute to a window of tumor vasculature normalization, e.g. by the reduction of VEGF-secretion, leading to reduced radiation resistance [319, 324, 325].

Overall, pharmacological-induced normalization of the tumor vasculature with concomitant reoxygenation is an interesting concept. However, a fractionated treatment regimen of IR with concomitant IR-induced reoxygenation might even supersede this effect. We currently cannot predict which tumor entities and phenotypes will respond to anti-angiogenic compounds accordingly, not even on the preclinical experimental level. Even more challenges exist in the clinics so that a defined window of tumor vasculature normalization could be identified on the individual patient situation, e.g. using hypoxia PET imaging, to be exploited for SBRT. Conflicting data on the outcome of different treatment schedulings exist when using IoAs in combination with IR. The studies suggest that it is not a neo-adjuvant but rather a concomitant and even more so an adjuvant treatment regimen that results in an enhanced tumor response to irradiation in combination with several investigated anti-angiogenic compounds [308, 309, 326–329]. As such, preclinical scheduling experiments suggest additional mechanisms to contribute to radiosensitization by anti-angiogenic agents, besides normalization of the tumor vasculature. IR affects the tumor vasculature in multiple ways, including the switch to other forms of angiogenesis [330, 331]. But irradiation also induces endothelial cell apoptosis, and the apoptotic response on the level of the tumor vasculature correlates with the tumor response to single high doses of IR (>15Gy) [332]. IoAs also induce endothelial cell apoptosis and thus might enhance the fragility of the tumor vasculature also to low dose fractions of irradiation and thereby increase the efficacy of radiotherapy [333–335].

#### Increasing Oxygen Delivery

Tumor hypoxia can be regarded as a shifted balance between demand and supply of oxygen relative to the normal tissue. Several earlier strategies were developed to increase the oxygen transport capacity of the blood and perfusion of the tumor with more oxygen. Hyperbaric oxygen (HBO) therapy [336] and blood transfusions in anemic patients [184, 337] failed to achieve a significant improvement in the outcome, which together with the complexity of the procedure and patient compliance led to a loss of interest into these approaches. An increase of the hemoglobin concentration could also be achieved by erythropoietin injections, but clinical studies revealed increased radiation resistance upon erythropoietin, which can be linked to radiation protective erythropoietin-induced signaling in certain tumor cells [337–339]. The approach with the most clinical success is the combination of carbogen (95% normobaric oxygen + 5% carbon dioxide) to deal with diffusion-limited chronic hypoxia and nicotinamide to overcome acute hypoxia. Two clinical trials, BCON in bladder cancer [340, 341] and ARCON in HNSCC [342–344] reported improvements in the outcome.

Other approaches to modify oxygen delivery include allosteric hemoglobin modifiers (e.g. RSR13, that reached phase III trials, but failed to improve overall survival [345]), agents that improve the diffusion of oxygen (e.g. trans sodium crocetinate – TSC [346], currently investigated in phase III clinical trials in glioblastoma (NCT03393000), and oxygen transport agents based on hemoglobin and fluorocarbons (e.g. NVX-108 [347, 348], currently tested in phase II clinical trials in glioblastoma (NCT03862430)). The allosteric effector of hemoglobin myo-inositol trispyrophosphate (ITPP) was also shown to locally increase the pO_2_ in hypoxic tumors. Even though mixed results exists on the potency of ITPP, which is primarily due to pharmacokinetic aspects, we and others demonstrated its chemo- and radiosensitization capacity in both immunocompromised and immunocompetent tumor models [349–352], For a more comprehensive review on the topic, see [348].

#### Optimizing Radiotherapy Treatment Planning and Delivery: Dose Painting

Recent developments in the field of medical physics made advanced, highly conformal techniques of dose delivery, such as intensity-modulated radiotherapy (IMRT) and volumetric arc therapy (VMAT), a routine part of clinical practice [353]. Together with the development of real-time *in vivo* hypoxia-detecting methods such as FMISO- or FAZA-PET (see above), the concept of “dose painting”, which includes first identification of hypoxic regions and then a targeted increase in the dose to those regions, was established [354]. Although promising, such PET-guided hypoxia dose painting faces inherent problems derived from the spatiotemporal heterogeneity of tumor hypoxia and the resolution limit of PET [355]. The large multicenter trial RTEP5 (NCT01576796), in which patients with NSCLC received a targeted dose increase in hypoxic regions identified by FMISO-PET, initially failed to demonstrate an improvement in the tumor control by dose painting [356]. However, long-term follow-up showed an increase in the overall survival in the patients who received the radiotherapy boost [357]. Currently, the NCT02352792 trial is recruiting HNSCC patients and will investigate FMISO-PET-based dose escalation, with an interim analysis demonstrating the feasibility of the approach [358]. MRI-based identification of hypoperfused volumes is another strategy to define hypoxic areas in the tumor; such an approach is used in an ongoing clinical trial that investigates targeted dose escalation in HNSCC (NCT02031250). In contrast to the conventional dose-boosting strategies described above, Tubin et al. have taken an innovative approach to partially irradiate only hypoxic segments of bulky tumors, with a short course of hypofractionated radiotherapy [359, 360]. Based on the promising preclinical data, suggesting the occurrence of bystander and abscopal responses [361], and the success from the initial clinical experience [359, 360], this approach is now being tested in a phase I clinical trial (NCT04168320).

## Conclusion

A century of preclinical and clinical research on tumor hypoxia and its impact on ionizing radiation-induced cell killing have consolidated that a reduced pO_2_-level in the tumor represents a major hurdle for successful radiotherapy. The concept of fractionated low dose radiotherapy with concomitant iterative reoxygenation in between fractions and its clinical application for many tumor entities remain the most powerful but still insufficient approach to overcome hypoxia-related radiation resistance. Lack of successful pharmaceutical interventions and its integration into combined treatment modalities is not only due to the complexity of tumor hypoxia on its own but is also linked to the physics- and imaging-oriented discipline of radiation oncology and its strong focus on technical innovations.

Molecular, cellular and (patho-)physiological processes all contribute to a differential pO_2_ in the tumor in its complex dynamics during tumor growth and in response to treatment. Furthermore, invasive and non-invasive methodologies probe tumor hypoxia at differential spatiotemporal resolutions, and only complementary approaches characterize the hypoxia status of an individual tumor sufficiently. It is not surprising that only a few pharmaceutical approaches have reliably proven to decrease tumor hypoxia, rendering personalized strategies even more difficult. At the same time, radiobiological research during the last decades demonstrated that different treatment regimens influence blood perfusion and oxygen diffusion in a differential way. However, we only start to understand these processes on the mechanistic level to optimally increase the efficacy of radiotherapy in combination with clinically relevant hypoxia-modifying agents.

Radiotherapy is nowadays often combined with immuno-modulatory approaches resulting in improved locoregional control and overall survival for some tumor entities [116, 118–120]. However, tumor hypoxia not only represents a hurdle for successful radiotherapy but also creates an immunosuppressive environment [21, 117, 121]. As such, the development of potent tumor hypoxia-modifying agents and its integration into current novel treatment strategies in the field of radiotherapy holds great promise to further improve tumor control, the therapeutic window and successful radiotherapy.

## Data Availability

Data sharing is not applicable to this article as no datasets were generated or analysed during the current study.

## Abbreviations

AMPK: adenosine-monophosphate-activated protein kinase
BOLD MRI: blood oxygen level dependent magnetic resonance imaging
CAF: cancer-associated fibroblast
CTGF: connective tissue growth factor
D2HG: D-2-hydroxyglutarate
DAHANCA: Danish Head and Neck Cancer Study
EGFR: epidermal growth factor receptor
EMT: epithelial-mesenchymal transition
ER: endoplasmic reticulum
FAZA: 18F-fluoroazomycin arabinoside
FH: fumarase or fumarate hydratase
FMISO: 18F-fluoromisonidazole
GLUT: glucose transporter
GSH: glutathione
HAP: hypoxia-activated prodrug
HIF: hypoxia-inducible factor
HNSCC: head and neck squamous cell carcinoma
HRE: hypoxia response element
IDH: isocitrate dehydrogenase
IGF: insulin-like growth factor
IMRT: intensity-modulate radiotherapy
IoA: inhibitor of angiogenesis
IR: ionizing radiation
ITPP: myo-inositol trispyrophosphate
LDH: lactate dehydrogenase
α-KG: α ketoglutarate
MRI: magnetic resonance imaging
NADPH: nicotinamide adenine dinucleotide phosphate
NF- κB: nuclear factor kappa B
NSCLC: non-small cell lung cancer
OER: oxygen enhancement ratio
OPN: osteopontin
OXPHOS: oxidative phosphorylation
PDGF: platelet-derived growth factor
PET: positron emission tomography
PHD: prolyl hydroxylase domain
PlGF: placental growth factor
PPP: pentose phosphate pathway
ROS: reactive oxygen species
RTK: receptor tyrosine kinase
SBRT: stereotactic body radiotherapy
SDH: succinate dehydrogenase
SPECT: single photo emission computed tomography
TCA: tricarboxylic acid cycle
TGF-β: transforming growth factor β
TPZ: tirapazamine
UPR: unfolded protein response
VEGF: vascular endothelial growth factor
VHL: von Hippel-Lindau
VMAT: volumetric arc radiotherapy
